# The Complex Journey of the Calcium Regulation Downstream of TAS2R Activation

**DOI:** 10.3390/cells11223638

**Published:** 2022-11-16

**Authors:** Maria Talmon, Federica Pollastro, Luigia Grazia Fresu

**Affiliations:** 1 Department of Health Sciences, School of Medicine, University of Piemonte Orientale, Via Solaroli 17, 28100 Novara, Italy; 2 Department of Pharmaceutical Sciences, University of Piemonte Orientale, Largo Donegani 2/3, 28100 Novara, Italy

**Keywords:** bitter taste receptors, calcium signaling, respiratory system, smooth muscle cells, epithelial lung cells

## Abstract

Bitter taste receptors (TAS2Rs) have recently arisen as a potential drug target for asthma due to their localization in airway cells. These receptors are expressed in all cell types of the respiratory system comprising epithelial, smooth muscle and immune cells; however, the expression pattern of the subtypes is different in each cell type and, accordingly, so is their role, for example, anti-inflammatory or bronchodilator. The most challenging aspect in studying TAS2Rs has been the identification of the downstream signaling cascades. Indeed, TAS2R activation leads to canonical IP3-dependent calcium release from the ER, but, alongside, there are other mechanisms that differ according to the histological localization. In this review, we summarize the current knowledge on the cytosolic calcium modulation downstream of TAS2R activation in the epithelial, smooth muscle and immune cells of the airway system.

## 1. Introduction

Type 2 taste receptors (TAS2Rs) are G protein-coupled receptors (GPCRs) first identified in the oral cavity, where they trigger bitter perception. The existence of a receptor family that is responsible for our ability to sense bitterness was predicted in 1995 by Lush and colleagues [1], but the coding genes and the respective proteins were only identified 5 years later [2]. TAS2Rs are expressed across the animal kingdom, but there exists a diversity among and within taxa in terms of the accumulated polymorphisms during their evolutionary process and the number of bitter taste receptor genes [3]. Fifty subtypes of TAS2Rs have been identified in mammals, of which 25 are in humans [4,5,6,7]. As expected, the different TAS2R receptors recognize different bitter compounds. Yet, the specificity usually associated with GCPRs cannot be taken for granted: some subtypes bind few chemically defined molecules, whereas others are broadly tuned [8,9,10]. Whereas bitter compounds/agonists vary according to their chemical structure and complexity, their ability to bind to one or more TAS2Rs relies on the presence of specific moieties or chemical groups [9] such as, for example, the δ- or γ-lactone rings that bind specifically TAS2R46 [8] and the isothiocyanates that activate specifically TAS2R38 [11]. Interestingly, bitter taste receptor subtypes that also show a high sequence homology might display a significantly different agonist profile [7], making it difficult to predict bitter compound recognition based on protein sequences. For example, TAS2R31, TAS2R43 and TAS2R46 share 85% of the gene sequence but respond differently to absinthin, aristolochic acid, denatonium and strychnine [12]. Experiments performed on chimeric receptors have shown that the C-terminal portion of the receptor determines agonist specificity [12]. Additionally, the same bitter compound can be recognized by different receptors, although the affinity among the different receptors for the same agonist can range from micromolar to millimolar in range [13]. Indeed, concentrations at which bitter ligands are recognized may vary considerably. For example, quinine, chloroquine and denatonium are effective on target cells at millimolar concentrations [14]. In contrast, more specific and less studied ligands, such as absinthin (TAS2R46-specific), are effective in the low micromolar range [15].

**Figure 1:**
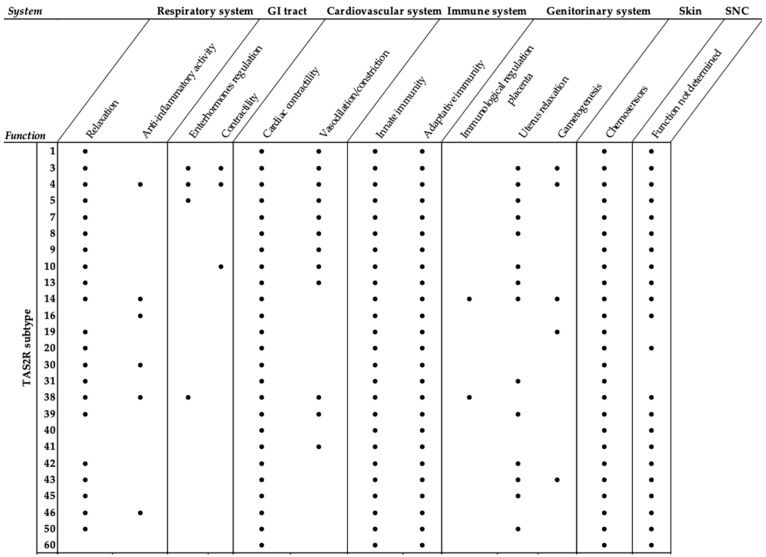
TAS2R expression and mediated functions in human tissues: respiratory system [14,19,26,27]; gastro-intestinal tract [20,31,32]; cardiovascular system [21,22,24,33,34]; immune system [26,35,36,37,38,39]; genitourinary system [29,30,40,41,42,43,44]; skin [37,45,46]; SNC [47,48].

From an evolutionary point of view, TAS2Rs are the most recent chemosensory receptors [3], appearing around 430 million years ago concurrently with the biological big bang and vascular plant expansion of the Cambrian period [16]. This simultaneous occurrence suggests that these receptors played a pivotal role in the evolutionary interaction between herbivores and plants [3]. Interestingly, it has been proposed that bitter taste receptors have appeared in the evolutionary process with primary roles different from the taste perception, such as nutrient sensing in the gut [3]. The hypothesis would be in accord with the more recent observations of the extra-oral localization of these receptors where they may be mediators of a wide chemosensory system [17,18]. Outside the oral cavity, TAS2Rs have been reported in the gastrointestinal tract, the brain, the heart, the immune cells, and the genitourinary and respiratory systems in which they exert different roles (Figure 1). TAS2Rs are involved in the regulation of the contraction/relaxation of muscle in different organs, such as the lung, gastrointestinal tract, heart, cardiovascular system and uterus [19,20,21,22,23]. The same subtype may be expressed in several organs and cell types at different levels of expression, mediating different physiological effects. For example, TAS2R1, when stimulated by dextromethorphan, leads to vasoconstriction in the pulmonary circuit and relaxation in the airway smooth muscle cells [24]. Moreover, bitter taste receptors create a wide net of defense systems throughout the body, being involved in both innate and adaptative immune response. TAS2R38, one of the most studied subtypes, is expressed in almost all the aforementioned systems. In the airway’s epithelial cells and macrophages, it acts as a sensor for bacteria and triggers an innate immunity response against exogenous pathogens with a well-described mechanism of action [25,26]. In particular, TAS2R38 detects the presence of the acyl-homoserine lactone (AHL) of quorum-sensing bacteria, and this leads to calcium-dependent NO production, which, in turn, stimulates cilia beating in the epithelial cells [27] and endows macrophages of cytotoxic activity [28]. Moreover, TAS2R38 has also been detected in the human placenta [29] with a hypothesized role in the immune regulation of mother–fetus communication and of embryo defense, using a mechanism that involves an intracellular calcium increase [29,30]. The heterogeneous distribution of TAS2Rs in ectopic tissues (Figure 1) was determined by the evaluation of both mRNA and protein expression, and their functionality was verified using cytosolic calcium release assays [17]. Altogether, these findings point out the complexity of bitter taste receptor signal transduction that cannot be simplified by describing the compatibility between an agonist and its receptor but depends on the anatomical localization, the specific cellular TAS2R patterns and the agonist response profile. In this review, we focus on the respiratory system, providing an overview of the current knowledge on TAS2R ligands and downstream cascades with particular attention to calcium homeostasis perturbation.

## 2. Bitter Taste Receptor Ligands

The human ability to perceive a countless number of bitter-tasting molecules with only 25 identified bitter taste receptors is an evolutionary achievement [6]. Bitter-tasting ligands (agonists and antagonists) are not only widespread in nature but are also widely diverse in chemical structures, which explains the necessity for receptor promiscuity [8]. TAS2R ligands belong to several chemical classes, including fatty acids; amino acids and peptides; amines and amides; azacycloalkanes; *N*-heterocyclic compounds; and other nitrogen compounds, such as carbamides, thioureas and ureas, alkaloids, terpenoids, glycosides and sugar derivatives [8]. The TAS2R agonist selectivity is underscored and regulated by several functional groups that characterize the structure of molecules. For example, human TAS2R16 and 38 are sensitive to compounds presenting the β-D-glucopyranoside moiety and thioamides [5,49], and TAS2R4 responds to denatonium benzoate and propylthiouracil, whereas TAS2R14 responds to the neurotoxic sesquiterpenoids (picrotoxin and picrotin) with a decreased potency of the latter [50]. Yet, the same receptor is also the target of substances with no evident structural correlation between them, such as α-thujone, naphthoic acid, piperonylic acid, 1-nitronaphthalene and 1,8-naphthalaldehydic acid, revealing its broadly tuned nature is shared also by TAS2R7, TAS2R10, TAS2R43, TAS2R44 and −47 [51]. Interestingly, natural compounds that help with the deorphanization of TAS2Rs belong to the chemical class of terpenoids and, in particular, to the four major classes of sesquiterpene lactones (germacranolides, guaianolides, pseudoguaianolides, eudesmanolides) and target TAS2R38 and TAS2R46 [52]. In particular, TAS2R46 represents the major bitter taste receptor for sesquiterpene lactone. The γ-lactone and δ-lactone moieties are critical for the binding, whereas the terpenoid skeleton and its functionalities play an important modulating role [51,53]. It is not surprising that the promiscuity that qualifies TAS2Rs can also be found in antagonist bitter molecules. This has so far hindered the discovery of selective ligands. For example, 4-(2,2,3-trimethylcyclopentyl) butanoic acid is an inhibitor of TAS2R44 but also an antagonist of TAS2R4, TAS2R7, TAS2R40, TAS2R43, TAS2R44 and −49 [8].

## 3. Bitter Taste Signal Transduction Mechanisms

**Figure 2:**
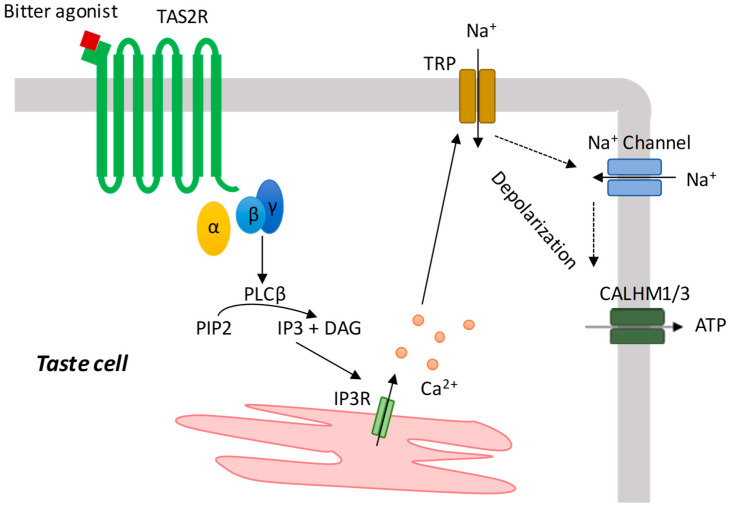
TAS2R signal transduction cascade in taste cells.

The observation that TAS2Rs are expressed across several tissues and cells has prompted the investigation of the downstream signaling cascades in terms of their physiological functions and possible pathological implications. The signal transduction mechanism following bitter taste receptors activation can be essentially divided into a first part, common to all cell types and a second part that differs according to the anatomical localization [54]. The common part of the cascade leads to calcium release from the endoplasmic reticulum (ER) via phospholipase C (PLC) mobilization [7,54] (Figure 1). Indeed, the ligand–receptor interaction determines a conformational change in the TAS2Rs, leading to the dissociation of the G protein subunits which trigger PLCβ2 activation. The enzyme thereafter cleaves the phosphatidylinositol 4,5-bisphosphate (PIP2) into inositol 1,4,5-triphosphate (IP3) and diacylglycerol (DAG) [23]. The binding of IP3 to its receptor IP3R on the ER determines the release of calcium and an increase in cytosolic calcium. In taste buds, the calcium elevation leads to TRPM5-mediated membrane depolarization, the release of ATP through CALHM1/3 and the activation of efferent nerve fibers, leading to taste recognition in the brain (Figure 2). In ectopic sites, the pathways downstream of the released cytosolic calcium are strictly dependent on the cell type in which the TAS2R resides.

## 4. Type 2 Taste Receptors in the Respiratory System

**Figure 3:**
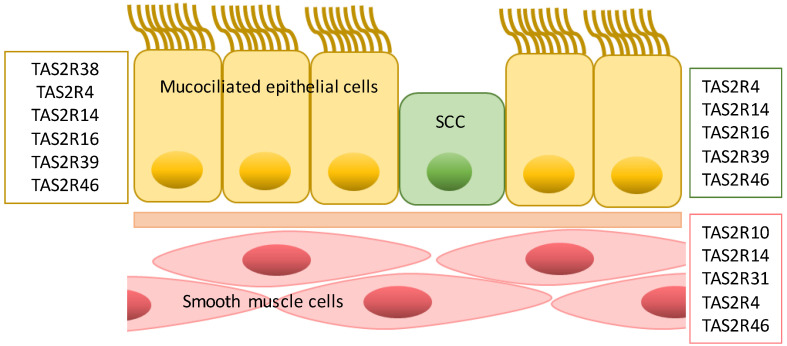
TAS2R expression pattern in airways cells. TAS2Rs expressed in mucociliated epithelial cells, in the solitary chemosensory cells (SCC) and smooth muscle cells are reported in the yellow, green and red boxes, respectively.

In the upper respiratory tract and lungs, TAS2Rs are expressed in airway smooth muscle cells (ASM), solitary chemosensory cells (SCC), epithelial cells and inflammatory cells [55]. Each cell type is characterized by a specific pattern of TAS2Rs and, as expected, the biological functions mediated by these receptors are different [55,56] (Figure 3). Bitter taste receptors in the respiratory system most likely are involved in restoring physiological conditions in diseased or irritated airways, considering the role they play in bronchodilation and airway clearance, as explained below.

### 4.1. Airway Smooth Muscle Cells

In ASM cells, the most represented bitter taste receptor subtypes are TAS2R10, TAS2R14 and TAS2R31. Their levels are abundant, and it has been suggested they are higher than the β2 adrenergic receptor [14]. Alongside these, attention has recently turned toward the less expressed receptor subtypes but which might be relevant from a pharmacological point of view, including TAS2R46 and TAS2R5 [8,15,19,57]. TAS2R46 is a receptor subtype expressed both in the airway epithelium and in smooth muscle cells, mediating anti-inflammatory and bronchodilating actions, respectively [15,58,59]. Structure–function studies classify TAS2R46 as a broadly tuned receptor. Indeed, both non-specific (e.g., strychnine) and specific (e.g., absinthin) agonists activate the receptor in the micromolar range [51]. This receptor subtype is the specific target of absinthin, one of the most potent bitter agents, with a bitter score of 30 µg/mL and a threshold value 6-fold lower than the minimal concentration required to activate TAS2R46-expressing cells [51,60]. The presence of TAS2R46 in smooth muscle and the availability of a selective agonist makes TAS2R46 attractive to study, as it is possible to explore its contribution to obstructive respiratory disorders pharmacologically.

**Figure 4:**
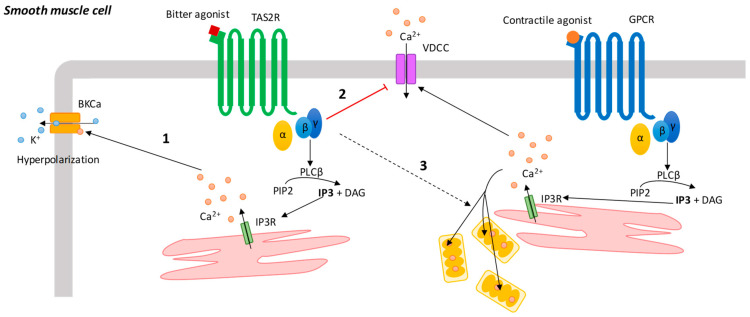
TAS2R signal transduction cascade in smooth muscle cells. 1. Cytosolic calcium increases open BKCa, leading to the outflow of K^+^ and, consequently, to membrane hyperpolarization. 2. TAS2R Gβγ subunit inhibits VDCC activated by a procontractile stimulus. 3. TAS2Rs promote the mitochondrial uptake of calcium released, following challenge with a procontractile stimulus.

It is well-established that TAS2Rs in ASM cells mediate the relaxation of precontracted cells but the mechanism by which this occurs is still controversial [14,61,62,63,64], as the calcium released from the ER represents a paradox considering the final effect. Indeed, contracting agents (e.g., histamine, endothelin 1 and carbachol) share the ability to induce cytosolic Ca^2+^ elevations [65]. Conversely, the calcium flux mediated by TAS2Rs is dose-dependently correlated to a reduction in the ASM cell traction forces. Starting from the seminal work of Deshpande and colleagues [14], several hypotheses have been made to delineate the signaling cascade of TAS2R activation in smooth muscle cells and how it leads to bronchorelaxation. The first obstacle to describe a unique mechanism is the absolute heterogeneity of TAS2Rs. Indeed, the downstream cascades invariantly depend on (i) the receptor subtype, (ii) the agonist–receptor binding and (iii) the contractile agent-challenging ASM cells. So far, the most evidence has been collected on TAS2R10, 14 and 31, as the most expressed subtypes. These subtypes are activated by millimolar concentrations of ligands, and this leads to an increase in cytosolic calcium, followed by membrane hyperpolarisation through large conductance potassium channels (BKCa) [14,61,66]. Yet, not all TAS2R agonists trigger a sufficiently robust cytosolic calcium increase to dilate smooth muscle cells. For example, absinthin and artesunate show dilating effects when used at micromolar concentrations but cause a cytosolic calcium perturbation only when used in the millimolar range [15,67]. In addition, other bitter ligands such as chloroquine, quinine and denatonium, instead of increasing the cytosolic calcium release, counteract it by inhibiting IP3 and reducing the Ca^2+^ sensitivity of murine ASM cells [68]. In addition to these observations, it is necessary to take into account that the amount of calcium influx is dependent on the level of TAS2R expression and the sensitivity of the receptor to the ligand [68]. For example, TAS2R46 is a poorly expressed receptor in ASM cells [8,14] compared to the most studied subtypes, but the high specificity and high affinity of absinthin allows the dissection of the role played by this receptor in bronchodilation [15]. In support of this evidence, Zhou and colleagues [69] recently demonstrated that the calcium increase following TAS2R activation is not sufficient to determine the phosphorylation of the myosin chain [69] and thus to trigger ASM cell contraction. Another relevant feature of TAS2Rs is their specificity and selectivity towards the contracting stimulus. Indeed, a bitter taste receptor subtype may be able to counteract the bronchoconstriction induced by a determined stimulus, although not counteracting the bronchoconstriction from other agents. Similarly, this may be true for cytosolic Ca^2+^ rises. For example, denatonium inhibits cholinergic stimulation [61]; absinthin counteracts calcium peaks induced by histamine but not by bradykinin (Talmon et al., unpublished data); chloroquine inhibits cytosolic calcium rises induced by histamine but not by endothelin-1, while it is exactly the opposite for aristolochic acid [63]. Thus, bitter taste receptors can (i) act independently from any other stimulus leading to a TAS2R-mediated cytosolic calcium rise and (ii) inhibit a GPCR stimulus-induced contraction and/or Ca^2+^ rise (Figure 4). In the first case, the signaling pathway is the same described for taste cells with a different fate for the Ca^2+^ release from the ER. Indeed, a localized calcium increase opens large conductance BK_Ca_, leading to the outflow of K^+^ and, consequently, to membrane hyperpolarization and cell relaxation [14] (Figure 3). It should be noticed, nonetheless, that other mechanisms have been proposed. Zhang and colleagues [62] proposed that the TAS2R Gβγ subunit mediates the inhibition of voltage-dependent calcium channels (VDCCs) activated by a procontractile stimulus [62] (Figure 3). Indeed, the authors demonstrated that in resting mouse ASM cells, bitter agonists determine a low-level increase in intracellular calcium via the canonical IP3 pathway, without affecting airway contractility. Yet, in the presence of a bronchoconstrictor stimulus, activated TAS2Rs lead to the inhibition of VDDCs, thereby leading to relaxation. This bronchodilating activity is impaired by inhibitors of the Gβγ subunit but not by IP3R antagonists or PLCβ inhibitors. Altogether, this also suggests that TAS2Rs might behave differently in resting or activated cells and that some of the controversies in the literature may be explained by this. It has been observed that TAS2R signaling interferes with the IP3-mediated contraction pathway, lowering cytosolic calcium concentrations to a level no longer sufficient to support the contraction [15,67,69]. For example, we have recently shown that the specific TAS2R46 agonist absinthin does not perturb intracellular calcium in resting ASM cells but instead counteracts the increase in cytosolic calcium when the cells are challenged with histamine. Absinthin does so by controlling and increasing the Ca^2+^ uptake in the mitochondria, making TASR4 the first GPCR able to control mitochondrial Ca^2+^ levels in this pathway [15] (Figure 3). It has also been demonstrated that TAS2R10 activation does not lead to any calcium increase in non-contracted smooth muscle cells, although in cells challenged by methacholine, it leads to cell dilation [67]. This has been suggested to be due both to a decrease in calcium oscillations and the reduced sensitivity of ASM cells to calcium release [67]. Altogether, these observations confirm the ability of activated TAS2Rs to impair IP3-dependent bronchoconstriction independently of the ability to activate PLC. Recently, another downstream signaling for TAS2R has been described in ASM cells co-treated with methacholine and kudinoside A (a specific ligand of TAS2R10): the bitter agonist inhibits the Gq recycling downstream of the acetylcholine receptor, resulting in the blocking of the signal transduction required for contraction [69]. All these observations, furthermore, underline the complexity of the interaction between a contracting agent and bitter ligand cascades.

### 4.2. Epithelial Cells

The most represented bitter taste receptor in ciliated respiratory epithelial cells is TAS2R38 which is activated by acyl-homoserine lactones (AHL) released by microorganisms and mediates the increased cilia beating to eliminate exogenous agents [27,70]. In solitary chemosensory cells (SSCs), other subtypes are expressed, such as TAS2R4, 14, 16, 46, 47, [71,72,73] and the very poorly studied 39 [74]. Overall, in these cells, TAS2Rs appear to have a common role in modulating the innate immune responses and counteracting inflammation [56,75]. Therefore, in epithelial cells, the bitter taste receptors act as sentinels of the airways, recognizing various pathogens, allergens or irritating molecules, such as the molecules sensitive to AHL quorum secreted by Gram-negative bacteria, and some molds, quinolones, formyl peptides associated with virulence [25,73,76,77,78]. Once recognition has taken place, the activated TAS2R leads to the release of cytosolic calcium from the internal deposits with the consequent release of cytokines, mucus and nitric oxide (NO) [59,75,79]. The stimulation of the Ca-calmodulin-dependent nitric oxide synthase induces an increase in NO [25,27] that, in turn, triggers, via cGMP, the protein kinase G which phosphorylates the cilia beating [80]. Most of the evidence of this cell type has been collected on TAS2R38, which is the most expressed and studied [25], and it remains to be ascertained whether all TAS2Rs perform the same role.

Indeed, other taste receptor subtypes have been shown to be expressed on the sino-nasal- and bronchial-ciliated epithelium, including TAS2R4, 14, 16, and 39 [25,81,82], and alternative transduction signaling ways have been described. For example, a subset of TRPM5 expressed in chemosensory cells has recently been identified that recognizes microbial peptides through TAS2Rs and responds through the canonical taste signal cascade. Moreover, TAS2R14 senses the competence stimulating peptide secreted by *S. Mutans* and triggers a calcium response through the canonical pathway Gβγ- PLCβ [83].

Another relevant issue in studying TAS2R localization and signaling is the differentiation stage of the cells. Indeed, McMahon and colleagues [84] recently analyzed the downstream calcium signaling in ciliated and de-differentiated airway epithelial cells demonstrating a difference [84]. Indeed, denatonium and flufenamic acid determine an increase in nuclear, instead of cytosolic, calcium in non-ciliated primary and immortalized epithelial cells [84]. This calcium spreads to the peri-nuclear region and is taken up by mitochondria, possibly initiating the apoptosis of de-differentiated cells. The different calcium signals in non-ciliated cells is strictly correlated to the delocalization of TAS2Rs from cellular membranes to the nucleus [84]. This observation remarks on again the complexity of studying bitter taste receptor transduction cascades that depends not only on the cell type but also on the localization within the cell.

### 4.3. Immune Cells

Bitter taste receptors have also been shown to be expressed in lymphoid and myeloid cells. In lymphocytes, the most expressed bitter taste receptors are TAS2R10 and 38, in particular in CD4^+^ T lymphocytes [38]. However, in macrophages [28], the transcripts of at least 16 different subtypes (TAS2Rs 3, 4, 5, 7, 8, 9, 10, 14, 19, 20, 31, 38, 39, 43, 45 and 46) have been detected. Both in resident and monocyte-derived macrophages, bitter agonists inhibit cytokine release [58] and cell migration [85,86] and stimulate reactive species release [61]. In mast cells, TAS2R3, 4, 10, 14 and 46 are well-expressed and are hypothesized to exert an inhibitory function on leukocytes, particularly by counteracting the release of histamine in IgE-activated cells [87,88]. Interestingly, TAS2R expression appears to correlate to an asthmatic phenotype. Indeed, TAS2R13, 14 and 19 are upregulated in the blood leukocytes of asthmatic patients [88]. Therefore, the correlation of TAS2R expression and function with asthma makes these receptors more attractive as new pharmacological targets for broncho-obstructive diseases.

## 5. Conclusions

Intracellular calcium homeostasis governs most cell functions, and the consequence of a Ca^2+^ rise depends on the cell type, upstream receptor activation and the condition of the stimulated cell. In the airways, Ca^2+^ it is a key mediator for signaling leading to contraction, cell metabolism, motility, differentiation and proliferation [89,90] and it is well known that altered Ca^2+^ signaling is critical for the development of the asthmatic phenotype [91]. Starting from these premises, finding targets to restore physiological calcium homeostasis in patients affected by pulmonary distress is paramount. In this respect, bitter taste receptors that mediate bronchodilation and reduce inflammation, antagonizing cytosolic Ca^2+^ rises, appear appealing targets for pulmonary disorders.

## Data Availability

Not applicable.
